# Supplementary material for: Comprehensive Annotation and Functional Exploration of MicroRNAs in Lettuce
Source: Front Plant Sci. 2021 Dec 24;12:781836. doi: 10.3389/fpls.2021.781836 (PMC8739914; doi:10.3389/fpls.2021.781836)
Supplement: Supplementary file 2 [file Data_Sheet_8.ZIP › GSM2230747.plot/Lsa-miRN1655_Lsat_1_v5_gn_1_89060.1_879_TPlot.pdf]

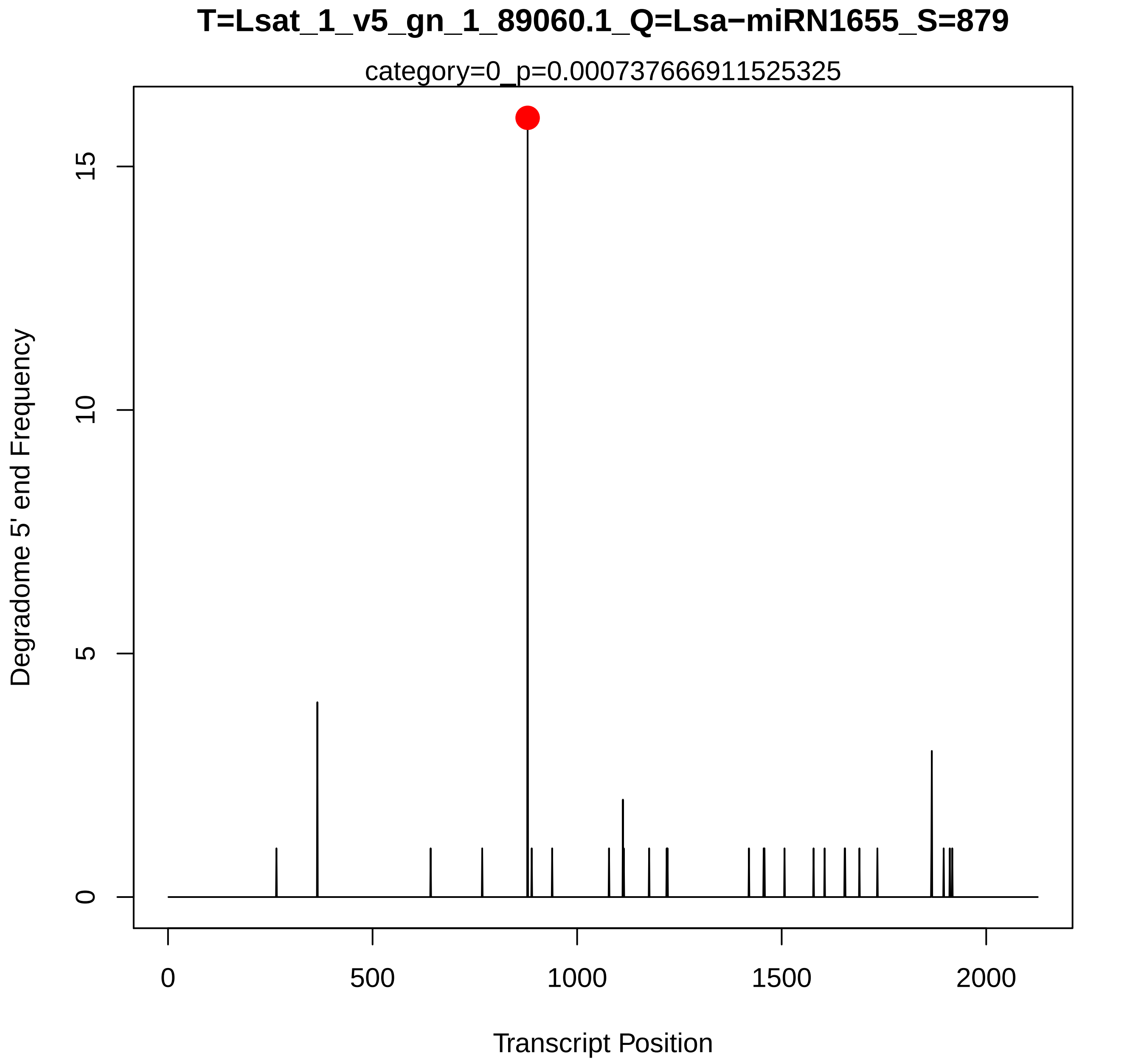

T=Lsat_1_v5_gn_1_89060.1_Q=Lsa−miRN1655_S=879
category=0_p=0.000737666911525325
Degradome 5' end Frequency
0
5
10
15
0
500
1000
1500
2000
Transcript Position
